# Raising a Public Health Concern: Women Overlooked in UK Drug Policy and Disadvantaged in Mixed-Gender Community Services

**DOI:** 10.3390/ijerph22101584

**Published:** 2025-10-19

**Authors:** Sarah Page, Fiona McCormack, Sophie Oldfield, Stephen Whitehead, Hannah Jeffery

**Affiliations:** 1 Centre for Crime Justice and Security, University of Staffordshire, LW118 Ashley 2 Building, Leek Road, Stoke-on-Trent ST4 2DF, UK; 2 Centre for Health and Development, University of Staffordshire, BL128 Science Centre Building, Leek Road, Stoke-on-Trent ST4 2AR, UK; fiona.mccormack@staffs.ac.uk; 3 Department of Social Work, Law and Criminology, C/O University of Staffordshire, Leek Road, Stoke-on-Trent ST4 2DF, UK; 4 Independent Researcher, London SE11 5DP, UK

**Keywords:** women’s health, mental health, drugs policy, alcohol, drugs, trauma treatment, domestic abuse

## Abstract

The British *From Harm to Hope* drugs strategy seems limited in gender responsiveness. Evidence is presented from a West Midlands case study where a qualitative participatory methodology with thematic analysis was employed. The project was co-designed by academics, women with lived-experience and third-sector research leaders. To identify community drug and alcohol treatment issues and solutions, interviews and focus groups were conducted with female service users (N = 28), a range of drug and alcohol workers and managers, and women’s sector practitioners (N = 17). Frontline professionals (N = 9) also took part in an online-adapted world café to enrich understanding and ascertain solutions. The study found that many women using illicit drugs have trauma and mental health issues linked to (1) adverse childhood experiences, (2) child removal by local authorities, (3) domestic abuse and sexually exploitative relationships, and (4) criminal justice system engagement. Based on findings, the study recommends that women’s public health pertaining to substance use, mental health and the interplay with childhood and adulthood abuse and violence needs better addressing in policy and practice. This paper highlights the need to better address women’s health through developing drug and alcohol services with improved referral pathways to domestic violence and mental health services.

## 1. Introduction

In 2021, British drugs policy [1] (*From Harm to Hope*) which was written by the Conservative party, endorsed an abstinence trajectory and aimed to cut drug-related crime, reduce supply and demand, and deliver quality recovery and treatment systems to drug and alcohol users. *From Harm to Hope* focused on getting more people into treatment and recovery, with additional finances to fulfil this ambition [1]. This short-term financial uplift was contingent on targets relating to treatment attendance and treatment completion being met. However, accessing drug and alcohol treatment in the UK may have gender disparities. For example, the Office for Health Improvement and Disparities statistics [2] demonstrate that substance treatment services are predominantly accessed by males, with approximately one third of those seeking services being female. With rising numbers of women with drug addictions [3], a possible treatment access gender inequalities gap is a concern. Health service access issues are on the agenda of the national Women’s Health Strategy published in 2022 [4] which aims to address several health matters, including reducing inequalities, improving women’s mental health, and reducing violence against women and girls. The interface between the women’s health strategy goals and substance use is not adequately covered in the national drugs policy [1], or in academic review on what is missing in the national drugs policy [5]. This paper endeavors to identify elements of what works for women regarding drug and alcohol community treatment services to inform service access and quality aspirations, particularly regarding improving mental health and reducing interpersonal violence.

Academics suggest that further amendments to the national drugs policy could address national and international evidence-base challenges to some approaches that it advocates [5]. For example, tougher sentences for recreational drug users could increase harm, due to people being deterred from acknowledging treatment need [5]. Women already show reluctance to seek help with intoxication issues due to fearing State child removal [6,7]. The drugs policy seems to promote tougher sentences, including incarceration, for recreational drug users [5]. However, there is limited prison drug treatment available [1], and criminal justice engagement increases stigma [7,8], which the British drugs policy is also aiming to address [1]. Fewer women may come forward for treatment with a punitive approach being employed that may result in forced child removal through incarceration. If wider policies were amended to focus on addressing health and social inequalities, removing children into looked after care (LAC) due to parental drug usage may be less needed, which may in turn reduce addiction issues and related parental suitability concerns [9]. Structures and inequalities fostering substance abuse are often overlooked [8,10]. By addressing inequalities in policy and practice, cost savings, particularly pertaining to reducing the need for numbers of children in LAC, could be made, furthering possibilities for greater success in addressing parental harmful substance usage.

Women’s healthcare is both a community and a prison matter, including drug and alcohol treatment. Inequalities for women regarding imprisonment are historically acknowledged [11], with women treated as ‘*doubly deviant*’ within the British justice system [12,13], including sentencing inequalities related to alcohol-induced violence [13]. Women with addictions are seemingly treated more harshly in the British Criminal Justice system and are often incarcerated further away from the family home than males [12,14]. This may impinge upon positive family connections, which are considered important for recovery capital [15]. Encouragingly, media reports document the now Labour government’s intent to reduce female incarceration [16], in favour of community rehabilitation and drug and alcohol treatment access.

Women accessing addiction treatment services experience gender stigmatisation, which we discuss extensively in a previous paper [7], particularly when women have childcare responsibilities, are pregnant, or undertake sex work [3]. Female street sex workers disclosing associated trauma encounter sexual advances from males in mixed-gender services and unwanted exploitative relationships; men become ‘*protectors*’ to encourage sex work to fund addictions [17]. There are additional vulnerabilities for homeless women engaged in ‘*survival sex*’ [18]. High prevalence of adverse childhood experience (ACEs) in the sex working community requires a trauma-informed healthcare approach [19]. Furthermore, safe accommodation could reduce vulnerability to sexual exploitation [18]. However, access to accommodation and treatment can be compromised due to organisational practices [7,18]. For example, some accommodation providers require abstinence and are mixed gender, which increases vulnerabilities to sexual exploitation. As discussed in our previous paper [7], women tend to use drugs and alcohol in harmful ways as a coping strategy to past and current abuses. Such early childhood abuses can seemingly set women on a path to multiple disadvantage, with poor access to services, resulting in unmet needs.

Drug and alcohol usage for women revolves around poor mental health and trauma experiences [3]. Early studies with justice involved women noted heroin usage to blank out childhood abuse, with associated crime as a poverty response [20]. Genderised abuse and exploitation continues as women and girls with ACEs, low income, and previous domestic abuse contexts are more at risk of being recruited into drug and sex trafficking work, leading to victimisation from severe sexual violence [21]. As such, male drug users can have a vested interest in a woman’s continued drug and alcohol use for exploitative purposes [7,21]. These abuses can be life-threatening, along with increases in drug-related death risks [22,23]. Female drug-related deaths were at 1803 out of the total 5448 deaths recorded by ONS in 2023 [24]. Alcohol usage and withdrawal can also lead to mortality [25,26], which has societal cost implications. Such costs to individuals and society give emphasis to the importance of improving female access to quality drug and alcohol treatment. Our research aimed to explore the lived experiences of women accessing community drug and alcohol services within the West Midlands region of Britain.

## 2. Materials and Methods

Our methodology is outlined in detail in a previous article discussing stigma [7]. Using a participatory ‘*collaborative*’ model [27], academics worked alongside students with lived-experience and third-sector partners to co-design and deliver qualitative research exploring women’s experiences of community drug and alcohol treatment services. Some researchers had service using history (such as attending talk therapy and undergoing alcohol and drug detox) and as such, they were lived-experience experts who were renumerated (through salaried positions combined with payment to the third-sector partner to provide gift vouchers) for contributions to the research team. Lived-experience experts were trained, or had prior training, in research and research assisting. Ethical approval from the University of Staffordshire, included British Sociological Association guidance pertaining to informed consent, anonymity, confidentiality, voluntary participation, sensitive questioning, debrief, and data protection [28].

A presentation to stakeholders across the West Midlands provided a self-selecting research sample with snowball links to other participants. Phase 1 was led by the Centre for Justice Innovation, a third-sector research and practice development organisation. This phase involved interviews and focus groups with a range of service managers and frontline staff from third-sector drug services and specialist services for vulnerable women (N = 17). Phase 2 was led by the University of Staffordshire and supported by Expert Citizens CIC. This phase involved semi-structured interviews and focus groups facilitated by academics and lived-experience researchers. Data was collected with women using services (N = 28), who were renumerated with GBP 20 gift vouchers. Support professionals from community treatment services or a women’s centre were available to the women during and immediately after data collection (N = 5, 2 of whom had lived experience). The interview and focus group questions were co-designed with women with lived experience and were included in the documentation submitted for ethical approvals. Participants received an information sheet and provided written consent to voluntarily take part in the study. Participants received a debrief sheet and verbal debriefed at the end of each data collection conversation.

Phase 3 was led by academics from the University of Staffordshire and enabled practitioners from drug services and women’s services (N = 9) to contribute experiences and solutions via an online-adapted world café with support from the wider research team. World café is likened to ‘*multiple focus groups*’ occurring at the same time, with rotation of group membership, whereby people will move to different discussion groups to contribute to the formation of a ‘*world view*’ [27]. World café is a participatory approach that aims to help participants to understand issues and to generate realistic solutions through people sharing experiences, knowledge and ideas [27]. At the online-adapted world café, which was facilitated on the Microsoft Teams platform, consent was gained, and an academic led presentation on the provisional findings from the study was shared using PowerPoint. Participants were then put into breakout groups facilitated by research team members with the presentation content stimulating the group discussions. These discussions were audio recorded and generated further data on experiences of service delivery to women and potential solutions to barriers to females accessing treatment [7]. The researchers fed back the main points from the group discussions when all participants reconvened in the main online meeting room. Participants were invited to bring their own refreshments to the online-adapted world café to maintain the spirit of hospitality.

All conversational data collected by the academics was transcribed verbatim from audio recordings and the six phases of reflective thematic analysis were utilised [29] using an inductive approach. The themes presented back to researchers by participants attending the online-adapted world café provided a participatory starting point for analysis. For online-adapted world cafés, interview and focus group data the research team employed the six phases of reflective thematic analysis: (1) dataset familiarisation through team discussions after data collection events, replaying audio recordings, and reading transcriptions; (2) data coding occurred as we started to note themes from the familiarisation process; (3) initial theme generation was activated as we started to colour code transcripts and add thematic notes; (4) theme development and review was achieved by the principal investigator reviewing the coding and discussing observations with the team; (5) theme refining, defining, and naming occurred through honing colour coding further; and (6) writing up findings for a round table event and a report to commissioners [30]. One salaried lived experience expert peer-researcher was trained and coached to code data using the reflective thematic analysis approach and worked alongside the academic principal investigator. The principal investigator is a feminist scholar who conducts research exploring the overlap between criminology and public health, working in these sectors prior to working in academia. Wider sense checking with lived-experience researchers occurred; such checks are valuable within the field of participatory research [18]. The principal investigator worked alongside a fellow academic co-investigator to finalise the articulation of the findings, providing opportunity for feedback from lived-experience experts. The interviews and focus groups with professionals were thematically analysed by the third sector partner. The analysis lead from the third sector partner is a feminist and subsequently has become a scholar within academia. Further sense-checking discussions occurred during phase 6 between the Centre for Justice Innovation and the University of Staffordshire project leads, and then wider stakeholders during phase 6 when fully drafted findings were shared with commissioners. For this paper, the primary data on domestic abuse and mental health was revisited, including re-reading transcripts and thematic note-writing iterations, with further discussion between colleagues at the University of Staffordshire and the Centre for Justice Innovation. A lived-experience expert researcher with coding responsibility from the project team reviewed the paper to ensure there was a final examination of how the data was being presented.

## 3. Results

This paper provides new insight into two inter-related themes linking to the women’s health agenda:

(1) Safeguarding concerns; domestic abuse and sexual exploitation exposure through mixed-gender treatment.
(2) Mental health and trauma needs; trauma treatment, and healthy relationship education being foundational to addiction recovery.

Wider themes are captured in a report [30], and a focus on the stigma that women experience when they engage with healthcare and criminal justice services is captured via a peer-reviewed journal [7], with specific criminal justice recommendations outlined in a practitioner article [31]. This paper focuses attention on safeguarding concerns in relation to mixed-gender drug and alcohol service delivery and on gaps in delivery that need to be better addressed through improvements to policy and practice utilizing previously unshared data. Furthermore, we identify a need for better mental health and trauma treatment service access and the importance of healthy relationship education to support ambitions of reducing violence against women and girls [4].

### 3.1. Safeguarding Concerns

In this paper, the term “*safeguarding*” is used to describe protecting women from dangerous men in community treatment services, and in the context of substance using, emotional, physical and sexual harms. It is also used to explore the role that such services can have in supporting women to understand interpersonal violence, and how to identify and respond to relational red flags. Furthermore, the term refers to the safeguarding of women when they were children, and with regards to when drug- and alcohol-using women have their own birth children. As such, safeguarding in this paper has a broad interpretation.

Women explained that it was difficult to avoid drug dealers in the community when entering, or leaving, drug services. Concern about this was heightened for those attending services with newborn babies due to their desire to safeguard their child(ren) and remain drug free. We perceive that drug dealers target people in recovery, attempting to keep them dependent on illicit substances for their own financial gain. Furthermore, one woman talked about her domestically violent ex-partner turning up at a women’s only treatment service, and how staff were able to take action to have him removed by the police due to a court order:

*“…cos he come outside, he would shout at me and things like that, and I told the staff, and he got arrested. He had a warrant as well* [in this instance she was referring to legal requirement to keep away from the woman, rather than a warrant]… *but it’s supposed to be a safe space* [referring to the treatment service]…”

(WWLE INTP2SP)

Essentially, this incident altered her perception of how safe the service was to attend, despite staff efforts to have him removed. On another occasion, this ex-partner followed her into a shop in the community:

*“… and I went into the shop and I said ‘please can I stay in here, I’m scared’ I said ‘I’ve called the police’ and they asked me to leave…”*

(WWLE INTP2SP)

She was horrified that the shop did not help a ‘*vulnerable*’ (sic) woman. Both drug dealers and domestically abusive ex-partners were easily able to track women down as they tried to access treatment and recovery services and navigated the wider community. Support services were unable, and not commissioned, to safeguard women in the wider community, where women spent most of their time. However, one gender-specific treatment service had daily activities (including art, music, and IT activities) for the women to engage with, which helped with avoiding drug dealers and users in the immediate community by providing a positive outlet for occupying time. A women’s centre also provided daily activities (courses, arts, crafts, and cooking, alongside talk-therapy and advice services) which the women welcomed for the same reasons. Conversely, most community treatment services only provided a once weekly appointment, without wraparound activities, leaving women feeling vulnerable in-between appointments.

Furthermore, women did not always feel safeguarded by recovery professionals in mixed-gender treatment settings. Some women experienced domestic abuse and sexual exploitation from male service users. Women alleged that their experiences were often diminished by ‘*toxic masculinity*’ (sic) viewpoints.

*“When we’ve been in mixed groups… it’s not just happened to me but what I’ve witnessed is a women will disclose something, ‘yeah and erm there was domestic violence and this happened and that happened’ and there will always be one man, ‘what about the men?’ (Group agreement)… like we get it, we know there’s violent women out there as well but we’re talking about women…”*

(WWLE G2P9)

*“Some of the things I wouldn’t have opened up in front of a bloke about…the drugs and stuff like that I would have done, but you know the violence and other stuff I wouldn’t have spoke about... They look down on ya, ‘why didn’t you leave him?’ Well, you don’t know my situation…”*

(WWLE G1P1)

Inappropriate comments from men created a barrier to women talking through details of past and current abuses that exacerbated drug and alcohol usage. Such comments were seemingly unchallenged by recovery staff. As a result, services were dealing with surface level conversations and not reaching the heart of the matter because women tried to guard themselves from toxic, unsafe, violent, and exploitative behaviour. Women described how some men in mixed-gender treatment settings lured women into domestically violent interpersonal relationships, which developed into sexual exploitation; women were groomed and coerced into sex work and/or shoplifting to fund both of their addictions.

*“… in a mixed-group, you might have a man come over to you and say ‘oh, you look nice today, do you wanna go out for a coffee?’, like an ulterior motive…”*

(WWLE G2P11)

*“I’ve experienced that in the rehab that I was in… it was a mixed* [mixed-gender rehabilitation center]. *And, I noticed quite a lot the men predatoring (sic) women that were really vulnerable, and like the affairs that were going on were unbelievable… and they* [men] *just preyed on the women.”*

(WWLE G2P7)

*“…it’s all very misogynistic (sic)… if they both start using together, you can often find the man will want the women to go into sex work to feed both their habits…”*

(WWLE G2P9)

Not all men behaved like this in mixed-gender treatment; a few were considered ‘*safe*’ and considered allies (sic). Women were understanding of males wanting to replace their drug or alcohol addiction with sex, as for some, this resonated with their own drives. Others reported filling the void with shopping and playing bingo.

Whilst women welcomed relationships to fill a void, they discovered that abusive interpersonal relationships negatively impact upon substance consumption levels. As such, professionals recognized the inevitability that a woman in recovery may fail to sustain the governments desired abstinence outcome [1] if engaged in a toxic relationship with someone from their mixed-gender treatment group, or with a partner not in services. However, abusive partners may want the woman to remain addicted to control them.

*“I’m going through a domestic situation with my partner… it’s very difficult to not drink cos it’s my go to…”*

(WWLE G1P3)

*“Cos my* [domestically violent] *ex-partner put it* [heroin] *in my coffee and I got addicted to it. And then I went to smoking it* [heroin] *and then I went to injecting it (SP—ok) And then I become clean* [abstinent] *3 years ago.”*

(WWLE INTP2SP)

A positive dimension to women-only services was that women became increasingly able to recognise that they were in a domestically abusive relationship. Talking openly about relationship dynamics with peers and female support workers enabled females to decipher abuse, particularly when the female was both a victim and a perpetrator of domestic abuse.

*“…Say they’re in a domestic violence situation… it’s that one moment that they just flip and they attack the partner… then obviously they come through to probation… aggravated assault or whatnot, but actually, there’s so much more background and so much trauma that’s happened to lead up to that one event.”*

(Specialist IP5)

Some professionals in treatment services understood this complex dynamic, but others failed to see the women as victims. Most women in our study talked about how professionals, including social workers, police officers, prison officers, emergency responders, and medical staff, communicated to them with victim-blaming views, lacking empathy [7], and women were rarely offered needed support services. For example, one global majority woman talked about the police turning up to a domestically violent incident after she had managed to wrestle a knife off her male partner, which he had threatened to stab her with. Instead of him being arrested, she was arrested for having the knife and behaving disorderly in the street where she had fled to from the house where she had been attacked in; she had been beaten up, had her life threatened, and now she was being criminalised. She went on to say:

*“… I didn’t know what I was experiencing was called domestic violence because I was that young, but I was just, you know, sort of classed as oh this is just a domestic* [referring to what the police called it when they intervened] *but I was never given any help. Would I have gone into a refuge? Who knows but I was never given the option.”*

(WWLE G2P9)

This participant talked about being a teenager when she first experienced interpersonal violence victimisation. Whilst there had been police involvement, no one explained the serious nature of the crime, or what support was available. As such, domestic violence became normalised as a ‘*domestic*’ (sic). Lack of awareness of the life-threatening crime of domestic violence and support available put her life further at risk.

For many women, healthy relationship modelling was limited; due to ACEs, they did not know what a healthy relationship looked like, which reduced capacity to spot signs of abuse and exploitation.

*“…being brought up around it* [drug use] *through family members being users and stuff like that… there was a lot of domestic violence at home, between my mum and my dad… the people that I hanged around with when I was older… it become a habit then* [using drugs] *and then I ended up getting addicted…I got into a relationship with erm, a man that was ten years older than me and he was a heroin dealer, which I wasn’t aware of at the time, and he was very heavily addicted to heroin… I never had a habit on it before I got with him, but then I still don’t point fingers at him cos I’ve got my own mind… I was in that relationship for seven years, so that addiction spiraled out of control. And then obviously, then sex work came into it from my addiction….”*

(WWLE INTP5FM)

In this situation, an older male was not transparent about his heroin addiction or associated drug dealing. Essentially, she was lured into this relationship under false pretences. He made heroin available to her and later engaged her in sex work to fund her addiction. This woman was a teenager at the time, and as such, this would be a child safeguarding concern, yet she felt responsible for her choices and less cognisant of the power imbalance in this situation and her choices were likely to have been manipulated.

Parents physically fighting, and in some cases taking illegal drugs, were the context in which drug use and toxic relationships dynamics were introduced and normalised. Children raised in this context seemingly had friendships and interpersonal relationships with older teenagers and adults, creating a power differential that influenced the girl’s/woman’s subsequent behaviour. Professionals also told us about situations where women’s sex and drugs education knowledge were seemingly experiential, involving coercion from peers, romantic partners, or parents/guardians:

*“… some of our women were in prostitution from the ages of 14... that’s through family pressure and often that’s because of the parents themselves needing money for drugs and alcohol…”*.

(Specialist IP2)

This professional talked about how women were sexually exploited by parents and other relatives to fund drug addiction. There is serious safeguarding concern pertaining to parents using drugs and proactively sexually exploiting their birth (or step) child(ren). That said, some women were raised in homes where parents were not engaged in illicit drug use, but were verbally abusive to them, creating an attention deficit that led to safeguarding issues and risk-taking behaviour.

*“… in my childhood so there was…a sexual abuse thing that happened when I was about six* [when on holiday]*… I would always say (inaudible) I had a really good erm childhood… but then looking back at it now… I really struggled to have a relationship with my Mum… my mum had quite openly said ‘I never wanted to have you’… I was brought up with the feeling of not feeling wanted, not feeling loved…She got quite nasty with her mouth and stuff… I had very low confidence and self-esteem, then I started getting into sort of risky behaviour because I was getting attention from that”*

(WWLE, INTP3FM)

In this instance, the birth mother had health conditions requiring prescription drugs that seemingly negatively impacted her personality. This participant talked about having sex as a teenager with multiple people when socialising in parks and when attending nightclubs underage. She would also steal cars for older children. These risk-taking behaviours were to gain ‘*acceptance*’ (sic) from peers due to the lack of positive attention from her birth mother. This is also likely to be due to the sexual violation experienced as a young child as per existing thinking [32].

ACEs were seemingly pivotal to future abusive interpersonal relationships and addiction. Professionals perceived that ACEs played a significant part in women’s drug and alcohol addictions. For some women, their addiction led to engagement in criminal activity [31], with stigmatisation from criminal justice and healthcare professionals as discussed elsewhere [7]. Of significant concern is that a small number of interactions with male professionals were also sexually exploitative [7]. Essentially, services can fail to safeguard women from exploitative and violent peers and unprofessional and abusive staff practice. Such failures in safeguarding have been lifelong; from parents not meeting their child’s needs through to how women were allowed to be treated in services and by professionals.

Women seemingly had knowledge gaps pertaining to their health and wellbeing; plausibly due to poor relationships with birth parents and/or being in LAC. One woman raised in a traveller community explained:

*“… you cannot tell* [referring to the traveller community] *about being on a period… we* [traveller community] *wouldn’t talk about drugs… basically not knowing what it was… my parents were alcoholics, but there were no drugs…”*

(WWLE INTP1SP)

In this instance, drugs education became somewhat experimental as her male partner introduced her to using substances. Her cultural context created barriers to accessing drug services, talking about personal matters needed to be with a female support worker based on her application of traveller community gender expectations.

We found that education gaps were filled through gender specific support services and associated group work conversations, and some women were also referred to educational courses. A healthy relationships course was accessible to women at a women’s centre and was regarded highly by the women who went on it. Such educational resources did not feature in most community drug and alcohol treatment services. One woman talked about embedding learning through multiple course attendance. She was highly motivated to remain abstinent from drugs to keep her newborn baby from being removed into LAC. She had significant trust issues with males and desired and requested women-only services.

However, such courses are not a panacea. Following course attendance, one woman described subsequently moving in with a new partner with a history of domestic abuse. She felt things might turn out differently for her because they were both currently drug-free.

*“I’ve done the freedom course a couple of times previously… I just met this geezer now a month ago and he has DV on his thing* [referring to Claire’s Law police disclosure of previous domestic abuse to current partners] *I have just been Probation, and they are like ‘oh…’ …they are just trying to separate us.”*

(WWLE, INTP28SP)

Interestingly, her mistrust of some professionals meant she viewed their concern as trying to put a stop to the relationship and happiness. During the 36 min interview, her partner phoned on multiple occasions to enquire where she was, and how much longer she would be. She responded to his calls; seemingly seeing this as normal and not a red flag. This woman communicated that she had poor mental health; occasionally feeling suicidal. She described struggling to get help at the points when she needed it. Taking drugs had been a distraction from memories associated to a previous abusive relationship when she had worked hard to juggle finances for both her and her abusive partners addiction, whilst raising children, who are no longer in her care.

*“for me, it is a distraction* [thinking of how to obtain drugs and taking drugs]*… I was still in an abusive relationship and maybe I didn’t want to face the reality… he used to lay in bed all day and I would go out* [shop lifting or sex working] *and I’ve got my 8-year-old looking after my 2-year-old…”*

(WWLE, INTP28SP)

More broadly, women told us that the trauma of child loss when their child(ren) were removed into LAC settings triggered further drug and alcohol usage [7]. Social workers were seen as “*already making their mind up in the first 30 seconds*” (WWLE INTP28SP) to remove children and not giving women sufficient chance to change their lives. Participant 28 described one social worker being upfront about what she needed to do to get her firstborn child back, and after one year of changes, she achieved her goal. However, her changes were not sustainable and subsequent social workers were less understanding, and children were removed from her care. She talked about social workers and housing support workers not having awareness about drug addiction and substitute medication, nor understanding why people take drugs. She perceived that when she was a child in foster care, that her social worker(s) did not understand how she felt, and why she behaved in an unruly manner which escalated to justice involvement. Essentially, women were highlighting that their trauma needs were not met as children in the LAC system, nor in their adulthood and that interactions with the State, including social workers, the police and healthcare workers, could be problematic, rather than supportive.

### 3.2. Mental Health and Trauma Needs

We reported from this study that professionals identified that “99%” of women had experienced trauma from ACEs and continued abuse from partners in adulthood [30]. This paper extends knowledge by providing an account of the types of ACEs women experienced and how this intersects with addictions, domestic abuse victimisation, and mental health issues. Most women articulated that drugs and alcohol were coping mechanisms for unmet trauma treatment needs:

*“Think it was trauma from my childhood, erm, not dealing with the past, erm, yeah and being sexually abused.”*

(WWLE IP22)

This participant described not being supported to process childhood trauma by the LAC system. One offer of support was made but at a time when she was not ready to process the associated emotional pain. Becoming a mother, seemingly heightened memories of the woman’s own childhood trauma and her alcohol and illicit-drug usage spiralled out of control. Other women recalled that trauma support was not available to them through the LAC system, nor in adulthood. As outlined in Section 3.1 of this paper, social workers did not seem to understand addiction, and the time and resources needed for someone to become abstinent and sustain recovery. We heard accounts that suggested that social workers disassociated themselves from the legacy of State failure to address the women’s trauma from ACEs and LAC, and their later adulthood domestic violence victimisation, sexual exploitation, child removal, and justice involvement. Concerningly, we also heard reports of social workers colluding with domestically abusive male partners with regards to childcare arrangements, this was reflected in favourable court decisions in which domestically violent partners gained parental custody over women trying to address substance misuse issues having made the proactive decision to leave the abusive relationship. It was seemingly overlooked that child(ren) had, in legal terms, experienced domestic violence victimisation [33] through what they had witnessed in the family home, and that the domestic abuse had been a trigger to the mother’s alcohol and/or drug usage. In such instances, the partner might weaponize the situation of legal child-care arrangements to further mentally and emotionally abuse the woman [34]. Essentially, for this woman in our study, it was mentally tormenting to know a violent person was now in control of her birth child(ren). State decisions over childcare arrangements seemingly caused emotional distress to women and put children and their mothers further at risk.

*“I was late because I came from, erm, social services* [regarding a childcare competence review] *and my* [domestically abusing] *husband doesn’t usually come, and he came. My husband and I.., We aren’t divorced yet. We’re separated, so he lives in the family home* [with their birth children] *I live in a erm, (inaudible) flat, which I hate, it’s very depressing… and I hate it (starts crying)”*

(WWLE FG2P4SP)

Given that most women talked about starting to drink alcohol and to use illicit drugs in their early teenage years, it seems that the significant matter of unresolved trauma from ACEs was not sufficiently explored nor therapeutically addressed at that time by the many professionals they interacted with from schoolteachers, social workers, healthcare workers, and criminal justice staff. While it may be that professionals assumed other professionals had made appropriate referrals for support, the reality is that trauma support is not readily made available to people who have been through significant abuses. This matter needs to be resolved given the government’s goal for increased people reaching abstinence [1].

Most women described uncompassionate and obstructive social work practice, and only one woman recalled being offered bereavement counselling to process grief from her children being removed from her care by the State. Child loss through State involvement was traumatic for women and this stimulated drinking and/or drug usage and a decline in mental health, reflecting the literature that child removal intensifies further adversity [35].

*P28: “I have three kids…he* [the second social worker in a series of social workers] *was awful, awful. I knew from the day he knocked on the door and I opened the door he just didn’t, that was it, he had made his mind up and I never got them back…*”

*Researcher (SP): and have you been able to get some support to process how you feel about that?*

*P28: “erm, I think it’s been hard because I have spent a lot of time and money* [using drugs as an escape] *to try to not think about that… But I did get letterbox contact the other day, it gets sent here* [women’s centre]*… coming off drugs…I feel miserable, and I want to die. I don’t want to die, I just don’t want to live every day like this… the guilt from you know from not having your kids and not being able to do it* [abstinence] *sooner. And not being able to get rid of that dickhead sooner. And not being able to get that time back again* [with the kids] *and most of all, they will probably hate me. And that makes me not want to be clean* [abstinent]*… I couldn’t get a diagnosis. I waited a year for an appointment* [mental health service name omitted] *and they said you need help and that, but because of the drugs and alcohol we won’t work with you”*

This woman describes poor mental health and guilt from not meeting the needs of her children, resulting in them being put into LAC. Essentially, this woman was rejected from mental health services because she had not been abstinent for a set amount of time. We heard on multiple occasions that it was difficult to be abstinent when childhood abuse or domestic abuse was playing through the women’s minds. Women indicated that it took several months through to “*three or four years*” to get mental health assessment and treatment appointments. We have previously expressed to criminal justice practitioners that with addiction to drugs and alcohol being of itself a mental health condition according to internationally credible mental health diagnosis tools, it should be a given that someone using drugs and alcohol in harmful ways can access mental health services, and concerningly this is not the case [31].

*Support Worker: See, I find that mental health will reject your referral if you’ve got dependency issues as well (P3—absolutely yeah) So it’s a catch 22 isn’t it? Which came first?*

*P4: When I ring the crisis team they always say, one of the first things they ask me obviously they’ll say ‘have you had a drink?’ (P3—yeah) But what they don’t understand is, if I didn’t have all this crap going on in my head I probably wouldn’t.*

(WWLE FG2P4SP)

Essentially, women who need mental health services to address root causes to their addictions are faced with barriers to getting much-needed support when in crisis. The reducing mental health issues for women intention in the national women’s health strategy [4] is seemingly not being fulfilled within the context of women who have experienced trauma and use alcohol and drugs as a coping mechanism. For the few women who were able to access mental health services, they mostly reported positive impacts from interventions. However, other systematic barriers occurred for women if they moved geographical location while waiting for their assessment. In focus group 2, women explained feeling devastated by the systematic challenges of getting support:

*P3: …last time I was in court, I was given an ATR* [alcohol treatment requirement] *and a mental health treatment referral, I’ve been on the waiting list for this that and the other for mental health and I understand the pressure and what, it’s a sorry state of affairs but, erm, I actually broke down and cried when I got the mental health treatment referral because I was relieved and I thought (P2—yeah) why did it have to get to this point to get the help I need?*

*P4: I was still on the waiting list for* [name of mental health hospital omitted], *it was before covid.*

*P3: Nearly four years, three years. And that was because I was put on a list that was two years before I even got, this was way back, erm, before I even got an appointment and then the appointment was with a psychiatrist and they said ‘no, you need to see a psychologist’ and I was chucked back into the system.*

*P4: I got my appointment with* [name of hospital omitted] *after waiting for so many years and then because I had moved to* [location name omitted], *they said ‘oh, we don’t cover that area anymore’ and I was like ‘I’ve got my appointment’, I think it was the day before I moved and they called from there to confirm my appointment and they said ‘no’, then I have to wait now for my next appointment.*

Despite women describing traumatic past and present experiences, post-traumatic stress disorder (PTSD) was seemingly missed in diagnosis. In Section 3.1, we outline multiple situations where women have experienced domestic abuse, which is trauma-inducing, and can, as demonstrated in research, include multiple head injuries [36]; yet we found that women were rarely diagnosed with PTSD or referred to a head injury specialist.

*“I suffer with PTSD, well it’s yet to be diagnosed…”*

(WWLE G1P3)

Women mostly recognized that their mental wellbeing was linked to ACEs, domestic violence trauma, child loss from State removal, as well as trauma from being justice-involved including incarceration. Interestingly, some of the women said they were diagnosed with personality disorder, rather than PTSD, when they finally had their mental health assessment after a substantial waiting period. This raised questions for us pertaining to whether women are getting the right diagnosis and right treatment opportunities. Few women in our study had swift access to counselling for past trauma, but one women’s centre project with in-house provision was seemingly having positive impact:

*“We have our own counsellors, so we don’t refer to the NHS as much (researcher—ok)… When somebody moves into* [the supported accommodation house] *they go straight to the top of the waiting list for counselling”*

(Professional supporting WWLE G3)s

We also noted other systematic challenges for women regarding accessing mental health and drug service treatment. Services lacked resources to persist in offering appointments to women if they did not attend sessions. Furthermore, there were professional assumptions that it had been a choice not to attend, whereas it may have been that a partner did not let her leave the home, or she felt mentally unwell and unable to leave the home [30]. We also found that support services may not be available at timely points, when a woman is motivated to address issues. Essentially waiting lists, multiple appointments in multiple locations with multiple services and unreasonable abstinence demands from mental health services seemingly impede upon women achieving recovery goals. In some cases, getting substitute drugs on a prescription as part of a therapeutic intervention to gradually reduce dependence had substantial waiting lists. In certain instances, professionals were uncomfortable to disclose that they were supportive of a woman being recalled into prison to access substitute drug prescriptions more quickly due to the waiting list in the community. Women retelling their trauma stories was considered problematic and joint working, assessment, and information sharing practices could reduce such issues [30].

Professionals perceived systematic service access issues occurring in terms of accessing addiction and mental health support. An impact is continued drug- and alcohol-related harms to the woman, including risk of death from drug overdose or drug- and alcohol-related illnesses [24,25,26]. There are crime-related harms to consider, particularly given that women ended up incarcerated and separated from family. Women not being able to access treatment and mental health support were also more likely to become a victim of crime, including re-victimisation. It also put women at greater vulnerability to crime perpetration.

*“… I’m supporting a lady now, who came out of prison, erm, she’s got schizophrenia… she’s got her mental health appointment... it’s taken three months for that to happen… we are having to try and get adult social care involved because there’s been a de-escalation, you know in mental and physical health… it could potentially lead to her, um, committing a further crime but through no fault of her own.”*

(World Café G1P2)

*“… we have our clients saying ‘we need help’ and we’re like ‘we have referred you’… I think that’s probably the biggest thing for us, isn’t it? Just the wait times (*G1P4*—yeah) for the mental health support…”*

(World Café G1P3)

*“…they turn back to the alcohol and the drugs because of the wait times because obviously it’s what they know and then they end up being re-exploited and you know, going missing or disengaging from service”*

(World Café G1P4)

Professionals felt helpless and frustrated regarding waiting times for necessary mental health support. The increased exposure to drug- and alcohol-related harm is costly to society in terms of State financial expenditure [1]. We argue that such delays in mental health service provision and in the addressing of unmet trauma needs are unnecessarily exacerbating financial impacts on the State. It impacts negatively on resources of addiction services and other support agencies who try to bridge the gap while women await mental health appointments. Essentially, the longer a woman is waiting for trauma support, the longer she will spend accessing services which fail to address the root causes of her drug and alcohol use, and the greater her likelihood of needing emergency services and justice involvement. Such services are expensive; costs could be reduced by facilitating swifter access to the kinds of support needed to address the root trauma causes. Instant access, at points when women are keen to engage, without the barrier of mental health services requesting abstinence for assessment and treatment, was wanted by professionals and women who use services. Projects providing a holistic offer to women with onsite multi-agency intervention was preferred by professionals in our study:

*“…community hubs, multi-agency working… where everybody’s under one roof… it’s a lot of economic benefit to it as well for councils and services… there needs to be everything from somewhere where there’s childcare…a warm welcome space, a clothes bank, foodbank, education, DWP support, you know, all of those things. It’s not as simple as just supporting women to be not involved in the substance misuse and the associations…”*.

(World Café G1P1)

There was voiced frustration that multi-agency hubs had not been fully actualised, and access to mental health services had not been resolved; both matters have been highlighted by practitioners for several decades. Professionals described how austerity had impacted service delivery effectiveness and best practice being implemented. This may explain why service gaps are evident in what the women told the research team. A lack of resources and, in some cases, recruiting the right staff to support women with addressing women’s mental health needs to be factored in challenges to service delivery.

*“…they only had male community practitioner nurses left* [following a female community psychiatric nurse leaving] *there had been kind of a dip in referrals because women predominantly liked to be supported by other women… then it’s waiting for them to recruit a new female CPN* [community psychiatric nurse]*… we’ve had to look at alternatives for referrals for mental health or getting them to go through their GP and all that, which can obviously be a slow process.”*

(World Café G1P4)

In this instance, delays in treatment occurred due to women not wanting to be referred to a male CPN. Reluctance to working with males seemingly stemmed from abuses experienced by male family members and intimate partners, alongside poor professional encounters and exploitation from males in mixed-gender services. In most cases, talking to males about traumatic life events was difficult. One woman described a positive relationship with her male support worker at a community drug service whilst acknowledging benefit from being in a women’s only residential unit linked to a women’s centre. Some women did not realise they could request a female worker, but others did. However, staffing resource and recruitment challenges were a barrier to accommodating such requests.

## 4. Discussion

Our findings identify safeguarding concern regarding victims of domestic abuse being put into drug and alcohol treatment services with predatory male domestic abuse and sexual exploitation perpetrators. This contravenes the Women’s Health Strategy [4] intentions to reduce violence against women and girls. Existing British research highlights that males in mixed-gender services have a higher probability of domestic violence perpetration than the general public [37], which presents a safeguarding issue for women in mixed-gender treatment. We question why domestic abuse victims are being put into drug and alcohol treatment and recovery groups where there is greater likelihood of there being perpetrators of domestic abuse and sexual exploitation? Past research highlights that re-victimisation can occur when female sex-workers are targeted by males within mixed-gender drug and alcohol services for exploitative purposes, hindering recovery [17]. Our research extends this knowledge, noting the targeting of women for exploitation is beyond this subsection of women in recovery settings. Any woman, and particularly the most vulnerable, can be at risk of becoming belittled, groomed, and exploited by males accessing the same services. Whilst some men were considered safe, others were dangerous. Across Europe there is evidence [38] that men in treatment and recovery may also be victims of domestic and sexual abuse, but that prevalence of such victimisation is more apparent for females who also have greater mental health needs than male counterparts.

Women reported having intimate personal relationships at a young age with older males; these males invariably introduced the girls to addictive drugs, and in some cases to sex work. Research in the UK has purported that women and girls are intentionally recruited by drug traffickers under the guise of a genuine interpersonal relationship for exploitative reasons, particularly women with ACEs and those who have been victims of domestic violence [21]. We discovered that parents and other relatives of young woman can also be the sex and drug traffickers, prostituting their children for drug money. Essentially, women in our study were failed by their families in being safeguarded against exploitative older males and by the State. Due to the apparent grooming strategy being used, women struggled to identify relationships of exploitation. Instead, they perceived they had choices, despite age power differentials and deception being used. Grooming experiences seemingly continue for adult women entering mixed-gender community treatment services, whereby males lure women into relationships, and then back into using drugs, coercing them into sex work, or other forms of crime to fund both of their additions. In some cases, the women perceived this to be a genuine love relationship, whereas others saw this as intentional exploitative practices from ‘*toxic males*’ (sic).

The existing evidence [17] and our findings highlight that mixed-gender services can thwart public health intentions for both drug recovery and reducing violence against women and girls. We admonish treatment services to better attend to safeguarding of women from sexual exploitation and domestic violence victimisation risks. This is particularly important given that research from Australia highlights that children raised in violent homes are more likely to experience domestic violence victimisation throughout their life course [39], and many women in our study reported repetitive cases of victimisation and had been raised in violent homes.

A woman in recovery may desire an interpersonal relationship for sex to fill the addiction void, and this can occur with increased risks to women in becoming exploited and violently victimised. We note that services need to better consider supporting women to make healthy choices on replacement activities, particularly when other activities, such as gambling, could become problematic to mental health and financial well-being and criminal justice involvement [40]. Few services in our study were for women only, with provision of wraparound activities to support women with positively filling their time and avoiding drug dealers and users in the community. Women in women’s only services conveyed that they found this better than previous experiences in mixed-gender community treatment.

Women having supportive conversations with female peers and trauma-responsive female support workers became enablers to drug and alcohol recovery. In a gender sensitive environment, women could more readily access healthy relationship education to help with understanding on what constitutes abuse. They were then able to frame any female domestic violence perpetration as a trauma response to previous victimisation. This was particularly important for women who were in interpersonally violent relationships where they were also violent towards their partner, often as a form of self-defence or learnt behaviour from watching their parents’ fight. Women were seemingly more able to untangle the nuances of relationship dynamics when peer support occurred. In contrast, we have previously discussed how men in mixed-gender treatment settings behaved in stigmatising ways towards female victims of interpersonal violence and minimised their experiences [7], despite some males in treatment also having abusive pasts [38]. This led to women shutting down in such settings, which is not conducive to the success of interventions to addressing the underlying issues of substance usage.

Women with past ACEs, especially those raised in violent households, had limited healthy relationship education, or drug and alcohol health education, lacking foundational knowledge and increasing vulnerability to exploitative and abusive relationships. Healthy relationship education is not offered to all women in treatment and recovery services, and yet so many have need for this intervention. Furthermore, some professionals may need educating on the nuances of abusive relationships to better understand the context that many women experiencing harms from substance use face. Some women accessing gender sensitive treatment were referred to a specific healthy relationship education course as a preventative measure to future abuse. The course enabled women to potentially identify relationship ‘*red flags*’ (sic) of toxic and abusive behaviours. Such education was viewed as helpful by women but was not a panacea. For example, one woman had done the course and later became intimately involved with a male in recovery who had a history of interpersonal violence. Education alone may not be sufficient to address learned behaviour patterns from repetitive toxic relationships, particularly if it is naively believed that being drug-free is the only enabler to desistence from perpetrating domestic abuse. However, others utilising the course started to put boundaries in place to protect themselves, such as requesting female support workers and community psychiatric nurses. Given that some professionals also sexual exploit women with lived experience of drug and alcohol usage [7], and the past abuses these women had experienced from males, it was understandable that preferences for female workers were being asserted. However, some women did not know they had this right, and some services could not fulfil such requests due to staffing capacity levels, delaying women’s treatment and recovery journey.

Despite the levels of interpersonal violence reported to us, in-depth trauma treatment options did not seem readily available for these women. Evidence from Australia shows that PTSD is a common symptom in survivors of domestic abuse [34], including for children who have survived living in violent households [41]. Women described to us having had experienced significant injuries, including head injury within interpersonal relationships, although head injury assessment referrals were not common [7]. Many of the women reported ACEs and continued trauma from adult interpersonal relationships; however, trauma treatment referrals were less common for female victims and survivors. Access to mental health services to address trauma was seemingly problematic. Previous research acknowledges barriers to people in recovery accessing mental health support [18,42] and drug and alcohol usage in women as a coping mechanism to previous abuse [11,20]. Our research concurred with these findings and concerningly flagged up a lack of mental health service provision for women with substantial waits to access diagnosis and treatment. Of particular concern is that mental health services create an unnecessary barrier to accessing services if the woman is not abstinent. Our findings note that wider professionals can lack empathy towards women using drugs and alcohol [7]. Most women endured mental health decline due to abuse but were unable to access mental health services due to waiting times and not being abstinent. Interestingly, some women had a diagnosis of personality disorder, but few had PTSD (post-traumatic stress disorder) diagnosis, despite having experienced trauma. As such, we question whether women are getting accurate diagnosis and appropriate treatment. Furthermore, underpinning research [42] to the national drugs strategy [1] highlights the need for combining mental healthcare into drug treatment and recovery [42], and our findings suggest that this aspect of the drugs strategy has not been realised for women in the community.

With recognition of legacy issues from unresolved trauma within LAC contexts and the levels of domestic abuse apparent in the cohort of adult participants in this study, we advocate that LAC settings need to model and teach healthy relationships to children; there is need for greater investment of resources to better support parents to learn how to have healthy relationships. Children in LAC are highly likely to need trauma treatment when able to process what has happened to them. Whilst previous research suggests that policy needs to address health and social inequalities to reduce the numbers of parents using drugs and alcohol, and in turn reduce the numbers of parents being regarded as unsuitable caregivers [9], we extend this further to posit that children in LAC need their trauma addressed by services to reduce the likelihood of needing drugs and alcohol as a coping mechanism for past abuse. In turn, this may help to reduce the intergenerational cycle of State removal and addictions in families. There seems to be a cycle where (1) children are removed from abusive households; (2) not facilitated to process their trauma, (3) then they self-medicate with drugs and alcohol; (4) end up in an abusive and exploitative relationship; and (5) and eventually become mothers themselves with the cycle continuing whereby their children end up being removed to LAC. This cycle needs breaking. A possible avenue for intervention is in how social workers respond to women who use drugs and alcohol. Recent research highlights that interactions between drug using women, and their social workers are stigmatising [43]. We found that with social workers behaving in stigmatising ways [7], it is hard for a mother to build a helpful working relationship with them. Furthermore, our findings raise concern that violent fathers can be sided with by social workers who seemingly do not appreciate the UK legislation that acknowledged that the child is a victim of domestic violence as well, even when the parent has not directly physically hit the child [33]. Mothers using substances as a coping mechanism to domestic violence and fleeing the relationship may need more support to become drug and alcohol free and to have custody of their children. The children will also need trauma support to process their own victimisation.

Greater emphasis in social care on resolving trauma and understanding the cycle of addiction in families could help to reduce the numbers of children going into LAC and the subsequent health costs from addiction and poor mental health. Social work practices could be amended to better support women unable to meet the needs of their children due to experiencing addiction and domestic abuse. Women with lived experience articulated that social workers did not seem to understand the nature of addiction and had unrealistic expectations. As such, we posit that training social workers to become more trauma informed with women who use drugs and alcohol would be beneficial along with training on the legislation pertaining to the victimisation from domestic abuse experienced by children. The nuances of such factors need to be better understood to inform policy and practice pertaining to child safeguarding and possible early and realistic interventions for families.

Our study generated a robust qualitative sample [44]; however, a self-selecting purposeful and snowball sampling technique was employed, which is not without bias. The sample was regional, and wider research in needed across the nation to gain further insights into the treatment experiences of women to address gender inequalities and improve health outcomes. That said, having presented the study at national conferences, our findings have resonated as nationally applicable. Further research is also needed to test findings that women’s only services seemingly produce better recovery outcomes for women, than mixed-gender treatment. Our research was focused on community treatment; as such, focused research into inpatient rehabilitation gender disparities is welcomed and for non-commissioned support, such as narcotics and alcoholics anonymous. It would be interesting to explore how women’s treatment in the UK compares to other countries. As such, a recommendation is for a comparative study detailing international literature on gender-sensitive and trauma-sensitive services combined with review of gender-informed practice in different drug policies in a range of nations to establish an up-to-date global public health perspective. As part of the global health understanding development on women using drugs and accessing treatment, lived-experience voices need to be paramount with primary data collection included.

## 5. Conclusions

To address the possible gender gap in accessing drug and alcohol treatment, we advocate for a gender-sensitive and trauma-responsive holistic service and further research into the outcomes of this will be needed. We found that women who were supported in women’s only services seemed to make recovery progress, and they perceived their changes to be more meaningful in this context, in comparison to when they have previously been in mixed-gender treatment. Our interpretivist qualitative findings indicate need for greater investment into women-specific drug and alcohol treatment provision and British drugs policy [1] revisions to include guidance on meeting gender-specific needs, combined with better linkages to the women’s health strategy [4] aspirations. One key safeguarding concern is that women can be groomed and sexually exploited in mixed-gender treatment by male service users, increasing the risk of being subject to further domestic abuse. Such continued abuse, combined with ACEs seemingly have negative mental health impacts on women. However, accessing mental health services when addicted presents a barrier, with women enduring long and inappropriate waiting periods before they can be assessed and treated for mental health conditions. A confounding issue is service demands for abstinence to receive mental health treatment, which women found difficult when navigating past trauma. PTSD was rarely diagnosed, and as such, it is plausible that women’s trauma is not being picked up by mental health professionals. As such, the women’s health strategy objective regarding improving women’s mental health [4] is not being met for women who use drugs and alcohol due to service access barriers. Black’s report [42] to the British government highlighted co-occurring conditions of addictions and mental health need addressing, with uplift funds aimed at service transformation. Our research evidence points to there being a system failure in addressing trauma and mental health needs for women who use drugs and alcohol, particularly for those who have been in LAC. Our findings highlight that professionals and women with lived experience of using services would prefer agencies to work together to deliver empathic, holistic, trauma-responsive and gender-sensitive services, alongside direct trauma and mental health treatment, combined with healthy relationship education. It would seem that women’s public health needs pertaining to addictions and the interplay with childhood and adulthood abuse, exploitation and violence, and mental health needs better addressing in policy and practice. Furthermore, women with ACEs are being somewhat failed in childhood in terms of (1) safeguarding children from abuse in the first place, (2) ensuring abused children can access trauma treatment, and (3) educating girls residing in LAC settings on healthy relationships. Girls and women often use alcohol and drugs as a coping mechanism to childhood abuse and adulthood domestic abuse and sexual exploitation. Our findings suggest that social care policy and practice concerning child safeguarding requires some improvement to better respond to meeting children’s trauma needs, and to be more realistic in terms of support packages provided to parents in need of drugs treatment, healthy relationship education, and parenting skills development. Social workers could benefit from training pertaining to domestic abuse and secondary abuse and recovery from illicit drug and alcohol use. Our findings show that women can make sustained changes when recovery support is gender-specific, trauma-sensitive, and includes support to break the cycle of domestic abuse. Essentially, policy and practice change across inter-related disciplines in the UK needs to happen if we are to more effectively reduce health related harms to women. Specifically, we observe possible amendment opportunities to the national drugs strategy and women’s health strategy so that positive health outcomes for women can be more readily achieved, particularly concerning reduction to violence against women and girls and improvements to mental health in conjunction with recovery from drug and alcohol harms. Our findings identify that professionals find it frustrating that such issues can be known for many years and yet are still not being addressed. Best practices identified by professionals, such as ‘*one stop shop*’ (sic) approaches to addressing multiple needs, are wanted and warranted based on our findings. Not improving services for women experiencing drug- and alcohol-related harms is putting women’s lives in jeopardy and increasing the likelihood of women becoming justice involved.

## Data Availability

Data for this study is unavailable due to privacy and ethical restrictions.

## Abbreviations

The following abbreviations are used in this manuscript:

LAC	looked after care
ACEs	adverse childhood experiences
ONS	Office for National Statistics
ATR	alcohol treatment requirement
PTSD	post-traumatic stress disorder
IT	information technology
GP	general practitioner
UK	United Kingdom
CIC	Community Interest Company
NHS	National Health Service
